# Cdk1 and SUMO Regulate Swe1 Stability

**DOI:** 10.1371/journal.pone.0015089

**Published:** 2010-12-06

**Authors:** Kobi J. Simpson-Lavy, Michael Brandeis

**Affiliations:** The Department of Genetics, The Silberman Institute of Life Sciences, The Hebrew University of Jerusalem, Jerusalem, Israel; Johns Hopkins University, United States of America

## Abstract

The Swe1/Wee1 kinase phosphorylates and inhibits Cdk1-Clb2 and is a major mitotic switch. Swe1 levels are controlled by ubiquitin mediated degradation, which is regulated by interactions with various mitotic kinases. We have recently reported that Swe1 levels are capable of sensing the progress of the cell cycle by measuring the levels of Cdk1-Clb2, Cdc5 and Hsl1. We report here a novel mechanism that regulates the levels of Swe1. We show that *S.cerevisiae* Swe1 is modified by Smt3/SUMO on residue K594 in a Cdk1 dependant manner. A degradation of the *swe1^K594R^* mutant that cannot be modified by Smt3 is considerably delayed in comparison to wild type Swe1. *Swe1^K594R^* cells express elevated levels of Swe1 protein and demonstrate higher levels of Swe1 activity as manifested by Cdk1-Y19 phosphorylation. Interestingly this mutant is not targeted, like wild type Swe1, to the bud neck where Swe1 degradation takes place. We show that Swe1 is SUMOylated by the Siz1 SUMO ligase, and consequently *siz1*Δ cells express elevated levels of Swe1 protein and activity. Finally we show that s*we1^K594R^* cells are sensitive to osmotic stress, which is in line with their compromised regulation of Swe1 degradation.

## Introduction

In *S. cerevisiae*, Swe1 (*Saccharomyces* wee1 homologue) inhibits mitotic Cdk1-Clb2 (Cdc28-Clb2) activity by phosphorylating Y19 of Cdk1, equivalent to Y15 of Cdk1/cdc2 in *S. pombe* and higher eukaryotes [1]. This modification is reversed by dephosphorylation by Mih1 (*S.p* Cdc25) [2]. Swe1 does not inhibit Cdk1 when associated with its cyclins Clb5 or Clb6, moderately inhibits Cdk1-Clb3/4 and strongly inhibits Cdk1-Clb2 [3]. When Swe1 is first synthesized in late G1 it is predominantly nuclear, but after bud emergence it is additionally localized to the bud-side of the mother-bud neck in an Hsl1 kinase, Hsl7 and septin dependent manner [4]. Hsl1 and Hsl7 are also required for Cdc5 (polo kinase) bud-neck localization [5]. Prior to its destruction in late G2, Swe1 is hyperphosphorylated by Cla4, Cdk1-Clb2 and Cdc5, all of which are present at the bud-neck [5], [6], [7], [8], [9]. Recently we have found that although Cdk1-Clb activity is essential for Swe1 destruction, the presence of Clb2 or its interaction with Swe1 is dispensable for Swe1 degradation [10].

Small Ubiquitin-related MOdifier (SUMO, Smt3, 17% identical to ubiquitin) is conjugated to its targets by a system analogous to ubiquitin. Smt3 is activated in an ATP-dependent reaction by thioester bond formation with the E1 activator Aos1/Uba2 [11], transferred to the E2 ligase Ubc9 [12] and passed to a substrate lysine, usually in the sequence ΦKxD/E, where Φ is a hydrophobic amino acid, and x is any amino acid. There are four SUMO-E3 ligases in *S. cerevisiae*; Siz1, Siz2 [13], Mms21 [14], and the meiotically expressed Cst9 [15]. Siz1 is responsible for the majority of vegetative growth sumoylation, with Siz2 conducting most of the remainder [13]. Despite Smt3, Aos1, Uba2 and Ubc9 being essential genes [11], [12], a *siz1Δsiz2Δ* strain is viable [13], albeit with a clonal lethality, manifested by a nibbled phenotype which is caused by the 2µ plasmid [16]. In contrast, *mms21Δ* cells are not viable, though mutations in the RING finger domain that abolish its SUMO-ligase activity such as *mms21* ^sp^, *mms21*-11 or *mms21* ^CH^ are not lethal [14], [17], [18], suggesting that Mms21 executes another, non-SUMO, essential function. Whereas other SUMO-E3 ligases are nuclear, Siz1 is additionally localized to the bud-neck [13], [14], [19], [20]. Many proteins have been reported to be SUMOylated with effects being substrate dependent but including ubiquitin mediated proteolysis and re-localization. Different types of proteins are known to be SUMO substrates, many of them are involved in DNA replication stress response.

## Methods

### Yeast growth, synchronization and manipulation

Yeasts were transformed by the frozen lithium acetate method [21] and are listed in Table S1. Plasmids used are listed in Table S2. Strains containing Cdk1^as1^, *cdk1^Y19F^* and *swe1^18A^* were kind gifts from D. Kellogg [6]. Strains in W303° lacking *hex3* or *slx8* were kind gifts from X. Zhao [22]. Strains in the JD52° background lacking SUMO E3 ligases were kind gifts from E. Johnson [13], [17]. Mutagenesis of plasmids to introduce K594R and K328R mutations into Swe1 was performed using the Stratagene Quikchange kit and verified by sequencing. Swe1 was tagged with 6myc using pRS306-S6M or pRS306-S6M-K594R cut with *ClaI,* or by using pRS405-S6M cut with *SnaBI*. Taggings and knockouts were confirmed by PCR. Standard Yeast-Peptone and synthetic media (pH 5.8) supplemented with the appropriate carbon source (2%) was used throughout. Cells were grown at 30°C. S-phase arrest and release was achieved by releasing cells from G1 arrest (growth to saturation) for 1 hour, adding 0.2 M hydroxyurea (Sigma) for 2 h followed by three washes with DDW and release into media containing 5 µg/ml nocodazole (Sigma). For growth rates of cells under stress conditions, OD_600_ was measured before and after 9 hours incubation in YPD with 0.75 M NaCl, 7.5 mM caffeine or water. For osmolarity experiments, cells were synchronized by two doses of alpha factor (5 µg/ml) and released for 1 hour prior to addition of 0.5 M NaCl. For pulse chases, Swe1-3myc was expressed for one hour following release from G1 arrest and glucose added to halt transcription, or was expressed for 1 hour in *cdk1^as1^* cells arrested in G2 with 0.5 µM 1NM-PP1 after which cells were released back into the cell-cycle. Cycloheximide was not added, to allow the cell cycle to continue.

### Immunological procedures and antibodies

Cells were harvested and killed using 20% TCA, broken with glass beads and extracted into 2× sample buffer. 10% acrylamide gels were used for SDS-PAGE. Antibodies used were mouse-anti-myc 1/1000 (a kind gift from M. Goldberg), rabbit-anti-Cdc5 1/200 (Santa-Cruz), rabbit anti-Clb2 1/500 (a kind gift from A. Amon), goat-anti-smt3 1/200 (Santa-Cruz), phospho-tyrosine 15 cdk1 1/1000 (Cell Signaling Technologies) which recognizes phosphorylated tyrosine 19 of *S.c.*Cdk1 (pY19), rabbit anti-phospho p38 1/1000 (Cell Signaling Technologies) which recognizes dually phosphorylated Hog1 (ppHog1), rabbit-anti-βactin 1/500 (Epitomics), rabbit-anti-Aco1 1/20000 (a kind gift from O. Pines) and mouse-anti-Tubulin 1/1000 (B512, Sigma). Secondary antibodies were from Jackson Laboratories.

For immunoprecipation, cells were released from G1 arrest for 1 hour and then 0.5 µM 1NM-PP1 (Toronto Research Chemicals) added for an additional 30 minutes. Cells were lysed in the presence of 10 mM NEM, yeast protease inhibitors (100×, Sigma) and yeast phosphatase inhibitor cocktails 1 and 2 (100×, Sigma). Extracts were incubated with mouse anti-c-myc (a kind gift from T. Ravid) overnight and with anti-Protein A beads for 2 hours before extraction into 2× sample buffer.

For Swe1-GFP experiments, Swe1-GFP was expressed for 90 minutes following release from G1 arrest and glucose added to halt transcription. Cells were immobilized using ConA on glass bottom tissue culture dishes (Mattek) and filmed using a Roper CCD Camera mounted on an Olympus IX70 microscope with a 60× oil objective.

## Results and Discussion

### Swe1 is SUMOylated on K594 in a Cdk1 dependent manner

Ubiquitin mediated degradation of Swe1 is one of the major modes of its cell cycle specific activity. The regulation of this degradation has been found to be exceptionally complex and dependent on a multitude of cellular inputs. Cdk1-Clb and Cdc5 activities are both required for destruction of Swe1, however interaction between Swe1 and Clb2 is dispensable [6], [10]. As Cdk1 activity is not required for Cdc5 to be active [5] we considered other potential mechanisms as to how Cdk1 regulates elimination of Swe1. The SUMO E3 ligase Siz1 is a Cdk1 substrate [23] whose stability is negatively regulated by the replication-fork poison hydroxyurea and by inhibition of Cdk1 [24]. We explored the possibility that SUMO is involved in Swe1 regulation.

**Figure 1:**
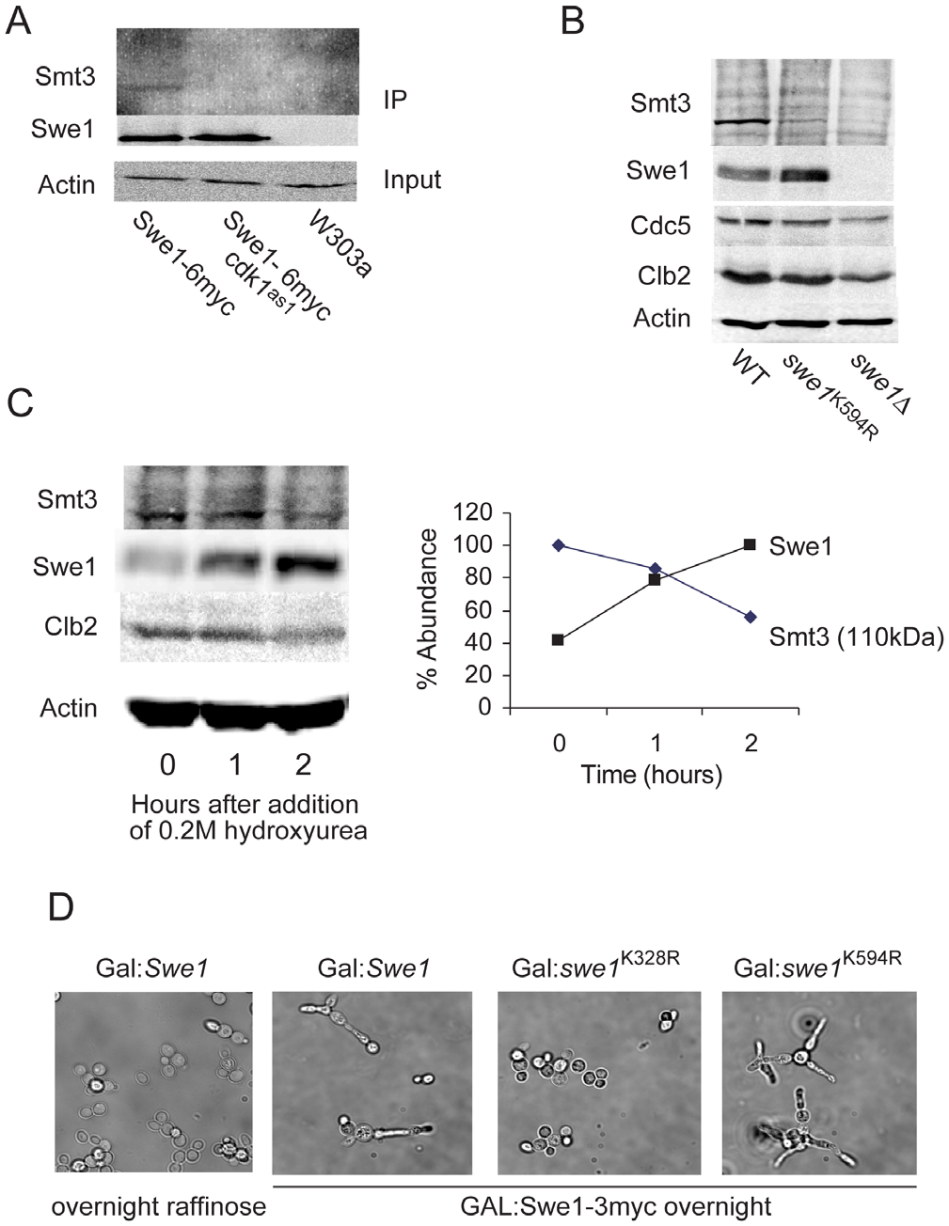
Swe1 is SUmoylated on K594 in a Cdk1-clb dependant manner. **A**. α-myc-Immunoprecipitation of Swe1-6myc cells shows Swe1 to be SUMOylated. Cells were grown for one hour before addition of 0.5 µM 1NM-PP1 for 30 minutes. SUMOylation is abolished when Cdk1 is inhibited. **B**. Whole cell extracts probed with anti-Smt3 show a SUMO band at 110 kDa (the size at which Swe1-6myc runs at) which is absent in *swe1Δ*, and Swe1^K594R^ cells. **C**. The 110 kDa Smt3 band is abolished by 0.2 M hydroxyurea. Quantification is relative to the maximum level. **D**. Morphologies of cells expressing wild-type and mutated Swe1 overnight from a GAL promoter. Wild type Swe1 and Swe1^K594R^ induced bud elongation (active) whereas Swe1^K328R^ does not (inactive).

Immuno-precipitation of a 6myc tagged Swe1 shows Swe1 to be SUMOylated (Figure 1A left lane). Tagged Swe1 was also precipitated from extracts of *cdk1* ^as1^ cells, which expresses cdk1 that is sensitive to the ATP analogue 1NM-PP1 [25]. In the presence of the inhibitor the precipitated Swe1 was not SUMOylated (Figure 1A middle lane), suggesting that sumoylation of Swe1 depends on Cdk1 activity.

Analysis of the Swe1 sequence with the SUMO site prediction program SUMOplot (Abgent) found a high probability (0.73) SUMOylation site at K594, which is within the Swe1 kinase-domain. This lysine is conserved from yeasts to man, with the SUMOylation site present in *sensu stricto* fungi (Table 1). Figure 1B shows that a prominent band of 110 kDa was detected when whole-cell extracts were probed with anti-Smt3. Strikingly this band was significantly reduced in extracts of cells expressing Swe1^K594R^ as their sole copy of Swe1 and almost absent from *swe1*Δ cells. Figure 1C shows that upon treatment with 0.2 M hydroxyurea, the levels of Smt3 at the 110 kDa band declines, whilst, as previously reported, Swe1 accumulates [26]. Together these observations suggest that SUMOylation of Swe1 on K594 is Cdk1-Clb dependent.

**Table 1:** Alignment of Swe1-K594 using fungal alignment on the SGD.

End		Start	Species	
614	ALRFIHDSCHIVHLDLKPANVMITFEGN**LKLG**DFGMATHLPLEDKSFENE	565	Sensu stricto	*S. cerevisiae*
624	ALRFIHESCHIVHLDLKPANVMITFEGN**LKLG**DFGMAAHLPLEDKSFENE	*575*	Sensu stricto	*S. bayanus*
610	ALRFIHESCHIVHLDLKPANVMITFEGN**LKLG**DFGMATHLPLEDKGFENE	561	Sensu stricto	*S. kudriavzevii*
618	ALRFIHESCHIVHLDLKPANVMITFEGN**LKLG**DFGMATHLPLEDESFENE	569	Sensu stricto	*S. mikatae*
614	ALRFIHDSCHIVHLDLKPANVMITFEGN**LKLG**DFGMATHLPLEDKSFENE	565	Sensu stricto	*S. paradoxus*
613	ALRFIHQTCHVVHLDLKPTNILITFEGN**LKLA**DFGMAAHLPLRDQDFENE	*564*	Sensu lacto	*S. castelii*
643	ALRFIHDSCRVVHLDLKPANVLITFEGT**LKLA**DFGMAAKLPISEEGFENE	594	Petite negative	*S. kluyveri*

The SUMO site is highlighted, identical residues in yellow and similar in green. However, mutation of G596A is predicted to abolish the SUMOylation site (Abgent SUMOplot).

We used the elongated-bud phenotype that occurs upon overexpression of wild type Swe1 [1] to determine whether the *swe1^K594R^* mutant could inhibit Cdk1-Clb2. Overnight expression of a single copy of *swe1^K594R^* from a GAL promoter resulted in elongated buds, similarly to overexpression of wild-type Swe1 (Figure 1D), indicating that this mutant is active. In contrast, overexpression of *swe1^K328R^*, whose mutation is in the region involved in interaction with Hsl1 [8] did not produce elongated buds.

### *Swe1^K594R^* cells express elevated levels of Swe1 protein and activity

**Figure 2:**
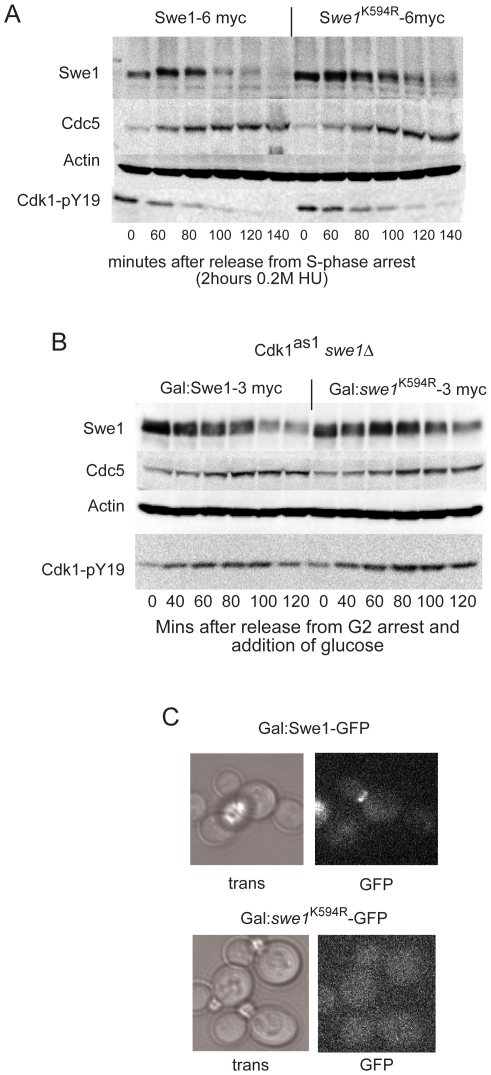
*Swe1^K594R^* is more stable and expressed in higher levels then wild type Swe1. **A**. Swe1 activity is more persistent with a delay in Cdc5 accumulation and destruction of Swe1 in *swe1^K594R^* cells. W303a cells were arrested for 2 hours with 0.2 M hydroxyurea and released into fresh media containing 5 µg/ml nocodazole. **B**. Swe1 activity is more persistent with a delay in Cdc5 accumulation and destruction of Swe1 when *swe1^K594R^* -3myc is expressed from a GAL promoter for 1 hour in *cdk1^as1^ swe1Δ* cells. Cells were released into media containing 5 µg/ml nocodazole and 4% glucose to shut-off Swe1-3myc expression. **C**. Localisation of Swe1-GFP and *swe1^K594R^*-GFP expressed from a GAL promoter for 90 minutes.

Wild-type and *swe1^K594R^* cells were released from hydroxyurea arrest into nocodazole (see [26]), and levels of Swe1 and Cdc5 as well as phosphorylation of Cdk1-Y19 were monitored. Figure 2A and Figure S1 show that *swe1^K594R^* cells displayed prolonged phosphorylation of Cdk1-Y19 and a concomitant delay in Cdc5 accumulation and Swe1 destruction. Pulse-chase upon release from G2 arrest of Swe1-3myc expressed from a GAL promoter as the only source of Swe1 in the cell likewise showed *swe1^K594R^* activity to be prolonged with a concurrent delay in Cdc5 accumulation and Swe1 destruction (Figure 2B). This novel mechanism to regulate Swe1 level could be one of the ways that Cdk1 regulates Swe1 destruction, since direct interaction and phosphorylation of Swe1 by Cdk1-Clb2 is not a prerequisite for Swe1 destruction [6], [10].

As bud-neck localization is involved in Swe1 destruction [4], [5], the localization of *swe1^K594R^*-GFP was examined. Whereas wild-type Swe1-GFP showed bud-neck localization prior to its disappearance, *swe1^K594R^*-GFP did not accumulate at the bud-neck and was diffuse throughout the cell (Figure 2C). It is thus conceivable that the elevated levels of *swe1^K594R^* and its delayed degradation are due to the lack of its targeting to the bud neck.

### The increased stability of swe1^K594R^ does not depend on its kinase activity or its interaction with Cdk1

**Figure 3:**
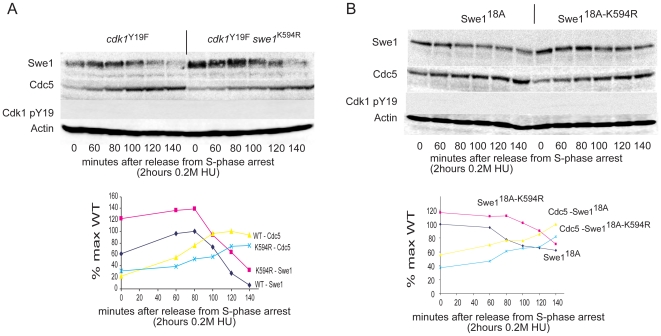
*Swe1^K594R^* abundance do not depend on its activity or interaction with Cdk1. **A**. *Swe1^K594R^* is intrinsically more abundant, even in a *cdk1^Y19F^* background. However, the delay in Swe1 destruction is abolished. Cells were arrested for 2 hours with 0.2 M hydroxyurea and released into fresh media containing 5 µg/ml nocodazole. **B**. *Swe1^K594R^* is intrinsically more abundant, even when all 18 Cdk1 phosphorylation sites present in Swe1 are mutated. Cells were arrested for 2 hours with 0.2 M hydroxyurea and released into fresh media containing 5 µg/ml nocodazole.

Increased Swe1 activity could delay entry into G2 and subsequent Swe1 destruction, but it is also possible for the increased, persistent Swe1 activity to be a consequence of higher intrinsic Swe1 levels in the *swe1^K594R^* mutant cells. Indeed, *swe1^K594R^* is present at higher levels than wild type Swe1 during log-phase growth (Figure 1B) or when cells are arrested in S-phase (Figure 2A). To determine whether *swe1^K594R^* levels are linked to Swe1 activity, *swe1^K594R^* levels were examined in cells expressing *cdk1* ^Y19F^, which lacks the Swe1 phosphorylation site on Cdk1. Figure 3A shows that in these mutants, in which Swe1 activity could not phosphorylate Cdk1, Swe1 levels remained higher in Swe1^K594R^ cells but without a delay in the initiation of its destruction.

To eliminate the possibility that Swe1 is affecting Cdk1 by binding to it we tested the effect of the K594R mutation on the accumulation of *swe1^18A^*, in which all 18 Cdk1 phosphorylation sites were mutated to alanine resulting in a loss of Swe1 kinase activity and lack of interaction with Cdk1-Clb2 [6]. Figure 3B shows that also in this case, *swe1^K594R^* was present at higher levels in S-phase arrested cells and again the initiation of its destruction was not delayed. Therefore, the increased stability of *swe1^K594R^* is independent of its kinase activity and its interaction with Cdk1-Clb2.

### Siz1 is the SUMO E3 ligase of Swe1

**Figure 4:**
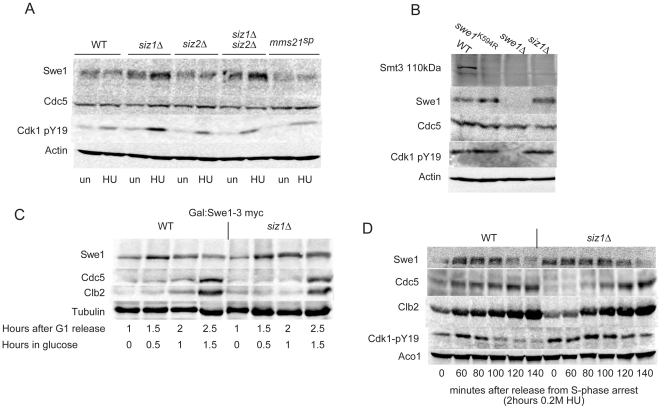
Siz1 is the SUMO ligase of Swe1. **A**. *Siz1Δ* cells exhibit elevated Swe1 levels. JD52° cells were left unsynchronized in mid-log phase (un), or arrested in S-phase with 0.2 M hydroxyurea (HU). **B**. The 110 kDa SUMO band (Figure 1B) is also absent in *siz1Δ* cells. **C**. Accumulation of Cdc5 and destruction of Swe1 is delayed in *siz1Δ* cells. Swe1-3myc was expressed from a GAL promoter in JD52° cells for 1 hour before being shut-off by addition of glucose. **D**. Swe1 activity is more persistent with a delay in Cdc5 accumulation and destruction of Swe1 in *siz1Δ* cells. W303a cells were arrested for 2 hours with 0.2 M hydroxyurea and released into fresh media containing 5 µg/ml nocodazole.

Of the three SUMO E3-ligases expressed during a mitotic cell cycle, the bulk of SUMOylation is carried out by Siz1 [13]. Levels of Swe1 in cells with knocked-out or inactive SUMO E3-ligases were examined under unstressed conditions or upon arrest in S-phase. Figure 4A shows that Swe1 levels and activity were elevated in *siz1Δ* cells. In contrast, Swe1 levels were not elevated above WT levels in *siz2Δ* or mms21^sp^ cells, even though mms21 deficient cells are hypersensitive to hydroxyurea [27] whereas *siz1Δ* cells are not sensitive [13], [28] (and E Johnson, personal communication). Further knockout of *SIZ2* did not significantly elevate Swe1 levels in *siz1Δ* cells. As expected deletion of the meiotic SUMO E3 ligase Cst9 also did not increase Swe1 abundance (Figure S3A). Figure 4B shows that indeed the 110 kDa SUMO band corresponding to Swe1 was absent in *siz1Δ* cells.

Pulse chase of Swe1-3myc in wild-type and *siz1Δ* cells shows Swe1 to be degraded slower in *siz1Δ* cells (Figure 4C). Similarly to *swe1^K594R^*, Swe1 levels were elevated in *siz1Δ* cells when growing in mid-log phase or when arrested in S-phase. When released from hydroxyurea arrest, *siz1Δ* cells showed a considerable delay in dephosphorylation of Cdk1-Y19 and a concomitant lag in Cdc5 accumulation and Swe1 destruction (Figure 4D and Figure S2). Although the effects of the Siz1 deletion could be through lack of SUMOylation of other proteins, the congruence of phenotypes with *swe1^K594R^* suggests Siz1 does indeed SUMOylate Swe1 on K594. Furthermore, Siz1 is the only SUMO E3-ligase that co-localizes with Swe1 at the bud-neck, suggesting that the bud-neck may serve as an organizing platform for components of the Swe1 regulatory pathway additionally to Hsl1/7, Cdc5 and Clb2. SUMOylation of Swe1 by Siz1 is thus a novel mechanism by which Cdk1-Clb regulates Swe1 stability.

A subset of SUMOylated proteins are ubiquinylated by Hex3/Slx8 or Ris1 Figures S3A and S3B) [29], [30]. Cdc5 accumulation following release from S-phase arrest was delayed in *hex3*Δ or *slx8*Δ cells, probably as a consequence of the sensitivity of these strains to hydroxyurea [30] but without affecting Swe1 abundance. This observation indicates that SUmoylation is unlikely to be required for Swe1 degradation per se.

### Swe1^K594R^ cells are osmosensitive

**Figure 5:**
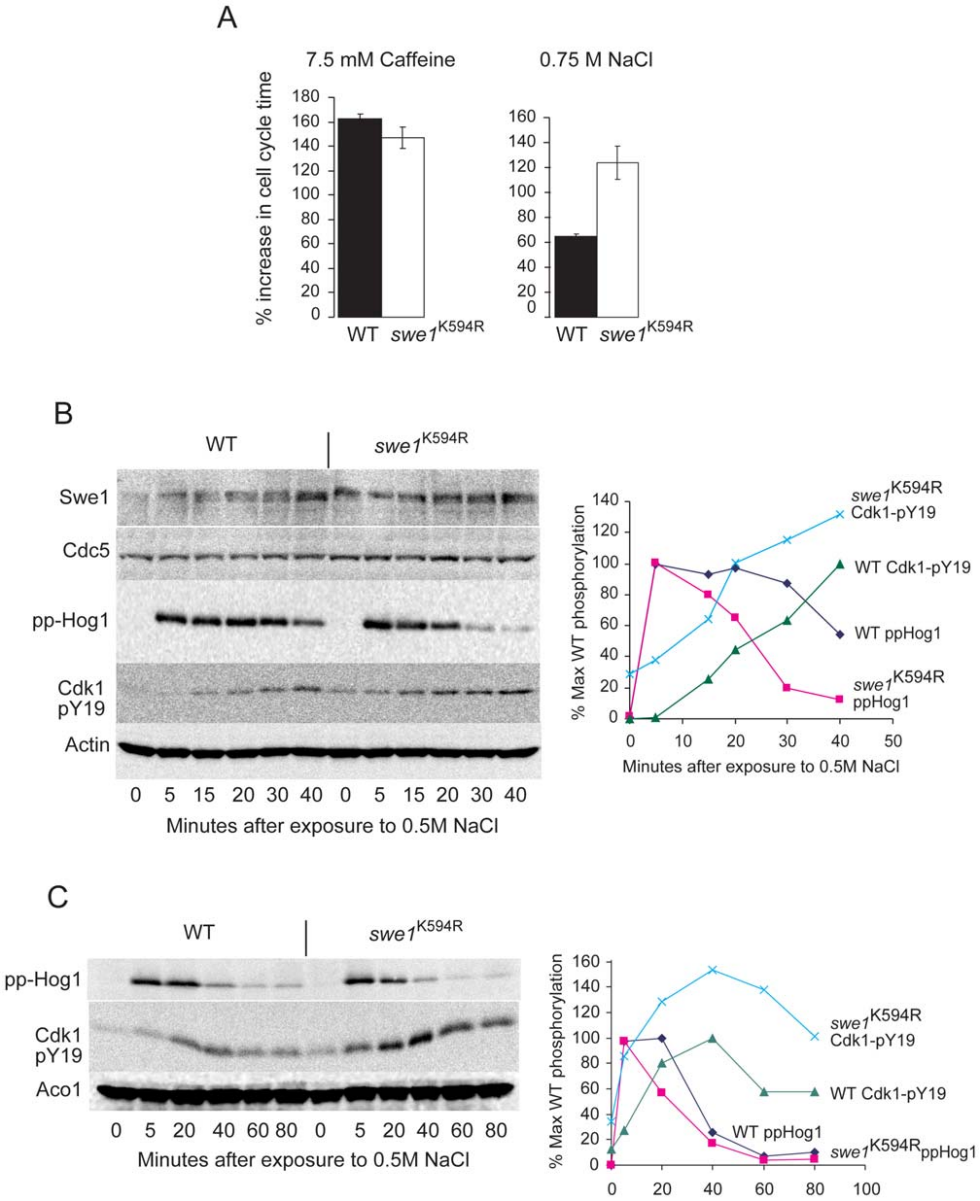
Swe1^K594R^ cells are hypersensitive to high osmolarity but not to caffeine. **A**. Growth rates of cells under stress conditions, OD_600_ was measured before and after 9 hours incubation in YPD with 0.75 M NaCl, 7.5 mM caffeine or water. For osmolarity experiments, cells were synchronized by two doses of alpha factor (5 µg/ml) and released for 1 hour prior to addition of 0.5 M NaCl. Growth error bars are two standard deviations from three independent experiments. **B** and **C**. Swe1^K594R^ activity is elevated and prolonged upon exposure to 0.5 M NaCl. Cells were synchronized in late G1 with alpha-factor (5 µg/ml) and released for 1 hour prior to addition of 0.5 M NaCl. The difference between the blots is the different time points.

Swe1 plays a central role in stress response and adaptation, consequently perturbations in Swe1 regulation often lead to stress sensitivity. We stressed wild type and *swe1* ^K594R^ cells with caffeine and with elevated levels of NaCl and determined their growth rate. Caffeine activates the cell-wall integrity (CWI) pathway and leads to dual-phosphorylation of the Slt2 MAPK [31]. Hyperosmolarity activates the Hog1 MAPK [32] pathway. Figure 5A shows that, in comparison to wild type cells, s*we1* ^K594R^ cells are sensitive to osmotic stress but not to caffeine. Cells were synchronized with α-factor, released into S-phase and the phosphorylation of Cdk1-Y19 and dual-phopshorylation of Hog1 monitored upon addition of 0.5 M NaCl. Phosphorylation of Cdk1-Y19 peaked at 40 minutes following stress, but was stronger and more persistent in the *swe1^K594R^* cells (Figure 5B and C). Hog1 was equally dually-phosphorylated after 5 minutes in both wild-type and *swe1^K594R^* cells, but dual-phosphorylation of Hog1 attenuated more rapidly in *swe1^K594R^* cells (Figure 5B and C). Together, this increased G2 delay and reduced Hog1 signalling duration could account for the osmo-sensitivity of *swe1^K594R^* cells.

Why would increasing Swe1 abundance through the *swe1^K594R^* mutation affect sensitivity to hyperosmotic stress but not to CWI stress? Whereas the only effector of hyperosmotic stress signalling upon Cdk1-Clb2 activity is through regulation of Swe1 destruction [33] CWI stress regulates Cdk1-Clb2 activity through multiple mechanisms [31] and thus the stress response is probably more buffered against increases in Swe1 abundance.

### Conclusions

Although Cdk1-Clb activity is essential for Swe1 destruction, activation of Cdc5 [5], Cdk1-Clb2 activity or interaction with Swe1 are dispensable for Swe1 degradation [6], [10]. We have found that Swe1 is SUMOylated on K594 and that this SUMOylation is prevented by inhibition of Cdk1-Clb activity or by knockout of *SIZ1*. Mutation of Swe1 to *swe1^K594R^* or knockout of *SIZ1* results in a greater abundance of Swe1 and consequently Swe1 destruction is delayed. Siz1 abundance is negatively regulated by hydroxyurea and inhibition of Cdk1 [24]. This provides a mechanism through which Cdk1-Clb activity indirectly decreases Swe1 abundance, and may serve to limit Swe1 levels during an unperturbed cell-cycle or following recovery from stress.

## Supporting Information

Figure S1 **Quantification of** **figure 2A** **.**
(TIF)

Figure S2 **Quantification of** **figure 4D** **.**
(TIF)

Figure S3 **Cst9, ris1, hex3 and slx8 do not seem to be involved in swe1 SOMOylation. A**. Deletion of *cst9* or *ris1* does not increase Swe1 abundance. Cells were released from G1 arrest (saturation) for 90 minutes, or synchronized in S-phase with 0.2M hydroxyurea for 90 minutes following release from saturation and then released into fresh media containing 5μg/ml nocodazole. **B**. Deletion of *hex3* (upper) or of *slx8* (lower) does not increase Swe1 abundance. W303°a cells were arrested for 2 hours with 0.2M hydroxyurea and released into fresh media containing 5μg/ml nocodazole.
(TIF)

Table S1 (DOC)

Table S2 (DOC)
