# Supplementary figures and images for: Cdk1 and SUMO Regulate Swe1 Stability
Source: PLoS One. 2010 Dec 6;5(12):e15089. doi: 10.1371/journal.pone.0015089 (PMC2997804; doi:10.1371/journal.pone.0015089)

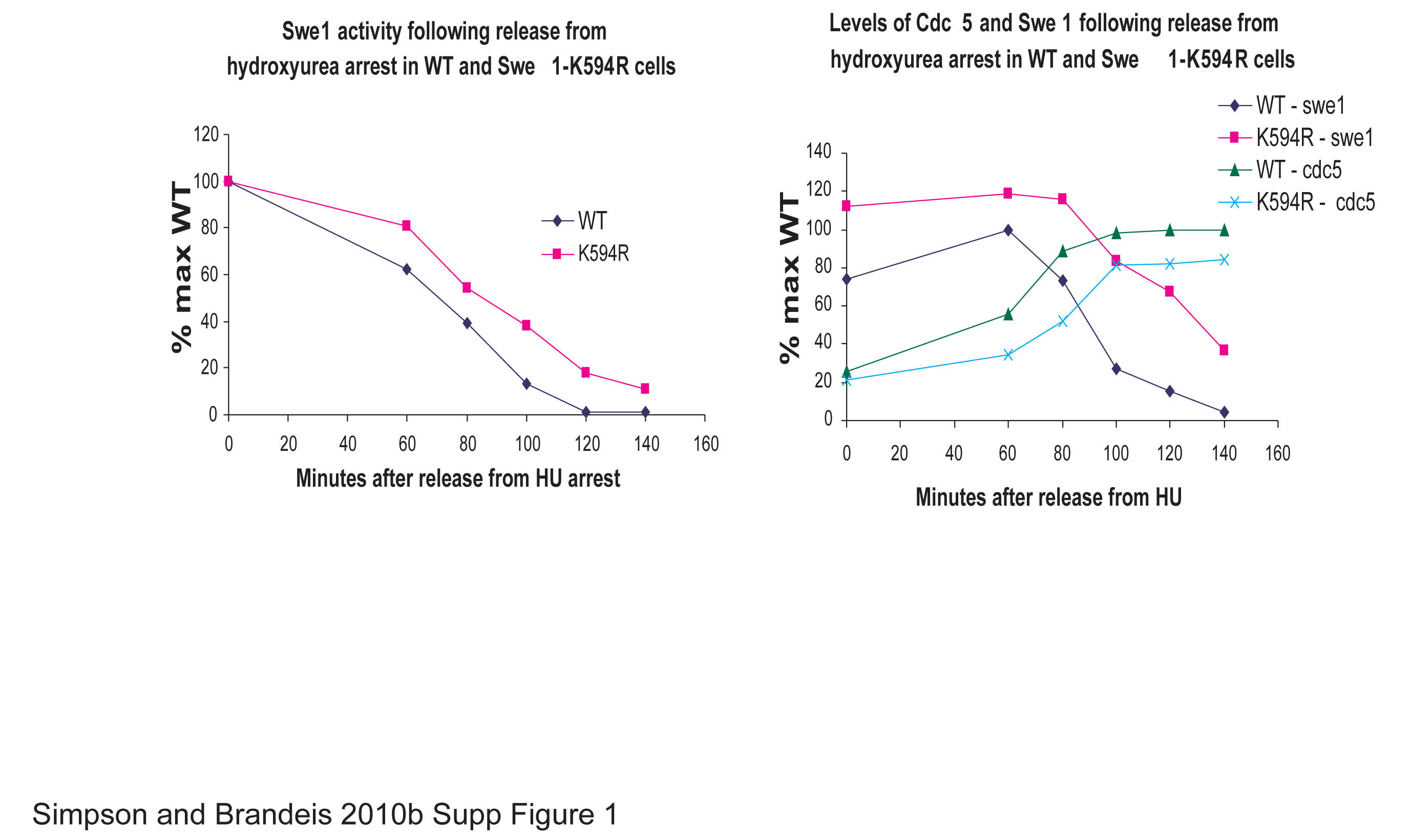

Supplement: Figure S1 — Quantification of figure 2A . (TIF) [file pone.0015089.s001.tif]

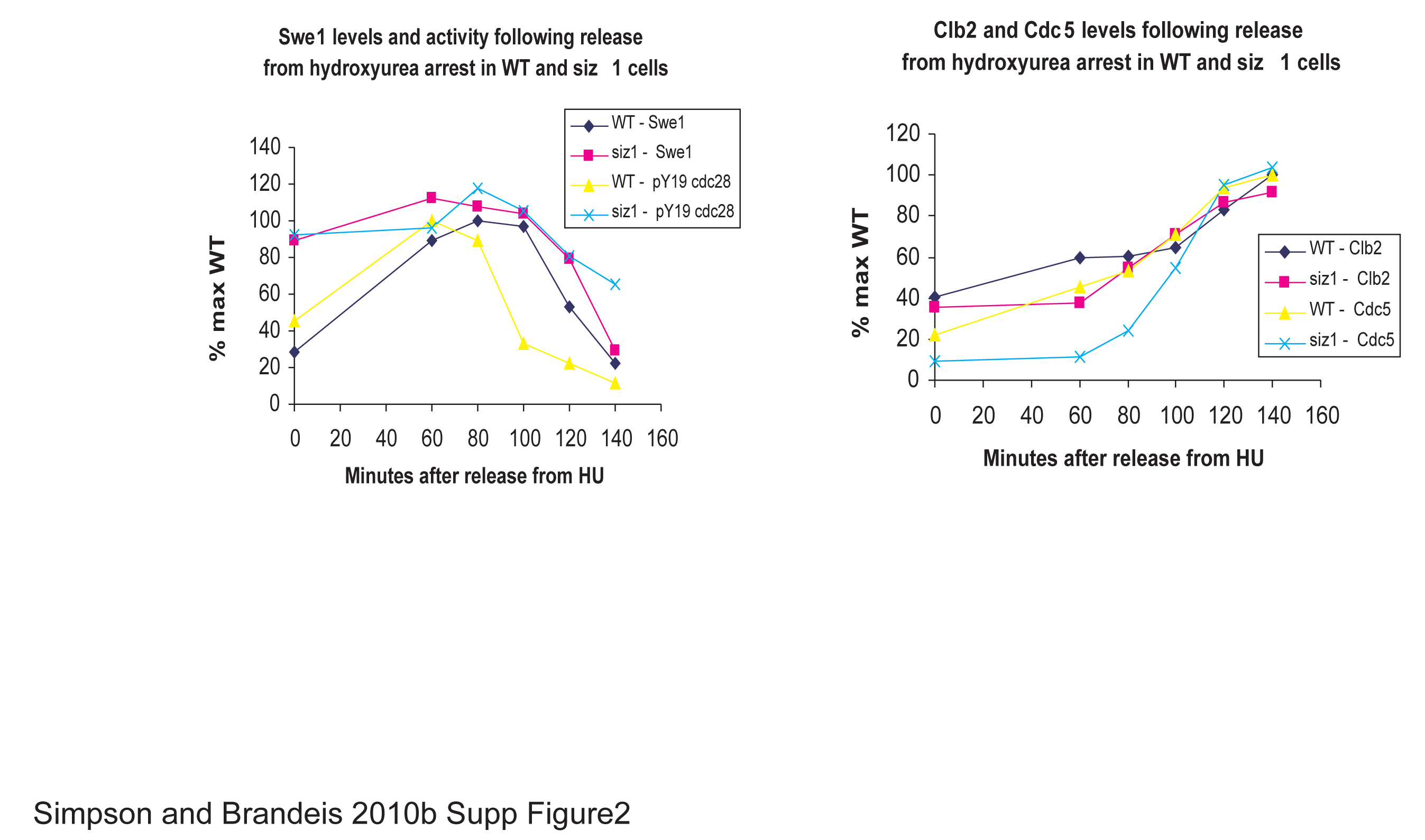

Supplement: Figure S2 — Quantification of figure 4D . (TIF) [file pone.0015089.s002.tif]

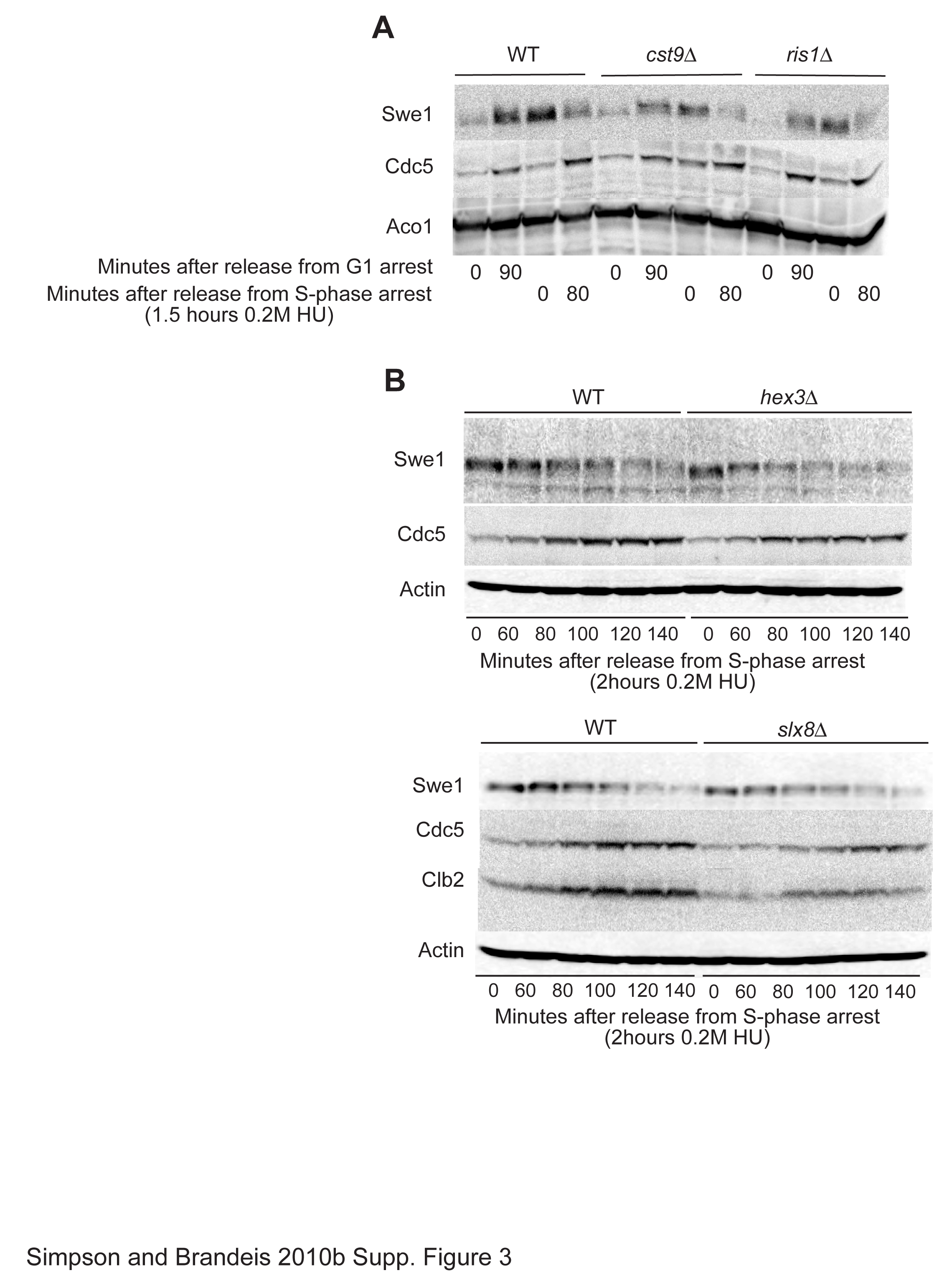

Supplement: Figure S3 — Cst9, ris1, hex3 and slx8 do not seem to be involved in swe1 SOMOylation. A. Deletion of cst9 or ris1 does not increase Swe1 abundance. Cells were released from G1 arrest (saturation) for 90 minutes, or synchronized in S-phase with 0.2M hydroxyurea for 90 minutes following release from saturation and then released into fresh media containing 5μg/ml nocodazole. B. Deletion of hex3 (upper) or of slx8 (lower) does not increase Swe1 abundance. W303°a cells were arrested for 2 hours with 0.2M hydroxyurea and released into fresh media containing 5μg/ml nocodazole. (TIF) [file pone.0015089.s003.tif]
