# Supplementary material for: Cdk1 and SUMO Regulate Swe1 Stability
Source: PLoS One. 2010 Dec 6;5(12):e15089. doi: 10.1371/journal.pone.0015089 (PMC2997804; doi:10.1371/journal.pone.0015089)
Supplement: Table S1 — (DOC) [file pone.0015089.s004.doc]

**Table S1** – **Yeast strains used in this study**

| Name | Description | Source |
| --- | --- | --- |
| W303a background | MATa ade2-1, his3-11, leu2-3,112 ura3-1, trp1-1, can1-100, rad5-535, | F. Uhlman |
| KS22 | swe1::SWE1-6myc LEU2 |  |
| JAU05 | Cdc28-as1 *bar1*Δ | D. Kellogg [6] |
| KS30 | swe1::SWE1-6myc LEU2 cdc28-as1 *bar1Δ* |  |
| KS38 | swe1::SWE1-6myc LEU2 cdc28-as1 siz1::URA3 *bar1Δ* |  |
| S6MK | swe1::SWE1^K594R^-6myc URA3 |  |
| SH24 | swe1::URA3 *bar1Δ* | D. Kellogg [6] |
| C28Dswe1 | Cdc28-as1 swe1::URA3 *bar1Δ* |  |
| S18A-6M | Swe1^18A^-6myc LEU2 |  |
| S18A-6MK | Swe1^18A-K594R^-6myc URA3 |  |
| Y19F-S6M | Cdc28^Y19F^-HA::URA3 swe1::SWE1-6myc LEU2 |  |
| Y19F-S6MK | Cdc28^Y19F^-HA::URA3::ura3 swe1:: SWE1^K594R^-6myc URA3 |  |
| S6M Dsiz1 | swe1::SWE1-6myc LEU2 *siz1*::URA3 |  |
| JD52º background | MATa his3-Δ200 leu2-3,112 lys2-801 trp1-Δ63 ura3-52 cirº | E. Johnson [13] |
| JD52-S6M | JD52º swe1::SWE1-6myc LEU2 |  |
| EJY325S6M | swe1::SWE1-6myc URA3 siz1::LEU2 | From EJY325 [13] |
| EJY324S6M | swe1::SWE1-6myc LEU2 siz2::TRP1 | From EJY324 [13] |
| EJY326S6M | swe1::SWE1-6myc URA3 siz1::LEU2 siz2::TRP1 | From EJY326 [17] |
| EJY392S6M | mms21-sp::URA3 swe1::SWE1-6myc LEU2 | From EJY392 [17] |
