# Supplementary material for: Cdk1 and SUMO Regulate Swe1 Stability
Source: PLoS One. 2010 Dec 6;5(12):e15089. doi: 10.1371/journal.pone.0015089 (PMC2997804; doi:10.1371/journal.pone.0015089)
Supplement: Table S2 — (DOC) [file pone.0015089.s005.doc]

**Table S2 -** Plasmids used in this study

| Name | Description | Cut with | Source |
| --- | --- | --- | --- |
| pRS405-S6M | Swe1-6myc in pRS405 LEU2 | SnaBI | O. Aparicio [3] |
| pRS306-S6M | Swe1-6myc in pRS306 URA3 | ClaI | O. Aparicio [3] |
| pRS306-S6M-K594R | Swe1-K594R-6myc in pRS306 URA3 | ClaI |  |
| gS3M | GAL:Swe1-3myc CEN TRP1 |  | Y. Wang |
| gS3M-K594R | GAL:Swe1-K594R-3myc CEN TRP1 |  |  |
| gS3M-K328R | GAL:Swe1-K328R-3myc CEN TRP1 |  |  |
| pMJS221 | GAL:Swe1-GFP CEN LEU2 |  | J. Thorner [5] |
| pMJS221-K594R | GAL:Swe1-K594R-GFP CEN LEU2 |  |  |
